# Open data could have helped us learn from another mining dam disaster

**DOI:** 10.1038/s41597-019-0063-0

**Published:** 2019-05-13

**Authors:** Paulo A. de Souza

**Affiliations:** Data61, CSIRO, 15 College Road, Sandy Bay, TAS 7052 Australia

**Keywords:** Industry, Environmental impact

## Abstract

There is an urgent need to improve integrity of large industrial infrastructure. Sharing data can support better understanding of accidents such as recent mining dam collapses, making them less likely to occur, and contributing to sustainability.

The recent Brumadinho dam disaster^1^ in Brazil is an example of infrastructure failure with catastrophic consequences. Over 300 people were reported dead or missing, and nearly 400 more were rescued alive^2^. The environmental impact is massive and difficult to quantify. The frequency of these disasters^3^ demonstrates that the current assets for monitoring integrity and generating alerting managers, authorities and the public to ongoing change in tailings are, in many cases, not working as they should. There is also the need for adequate prevention procedures. Monitoring can be perfect, but without timely and appropriate action, it will be useless. Good management therefore requires quality data. Undisputedly, management practices of industrial sites, including audit procedures, must improve, and data and metadata available from preceding accidents should be better used. There is a rich literature available about design, construction, operation, maintenance and decommissioning of tailing facilities. These include guidelines, standards, case studies, technical reports, consultancy and audit practices, and scientific papers. Regulation varies from country to country and in some cases, like Australia and Canada, it is controlled by individual state agencies. There are, however, few datasets available that are shared with the technical and scientific community more globally; particularly for prior incidents. Conspicuously lacking are comprehensive data related to monitoring of large infrastructures such as mining dams.

Today, *Scientific Data* published a Data Descriptor presenting a dataset obtained from 54 laboratory experiments on the breaching of fluvial dikes because of flow overtopping^4^. (Re)use of such data can help improve our understanding of fundamental processes underpinning industrial infrastructure collapse (e.g., fluvial dike breaching, mining dam failure), and assess the accuracy of numerical models for the prediction of such incidents. This is absolutely essential for better management of floods, mitigation of dam collapses, and similar accidents. The authors propose a framework that could exemplify how data involving similar infrastructure can be stored, shared, published, and reused.

## Trade-offs from Open Data

Industries could benefit from a common approach to store and publish datasets. Improved audits and due diligence, and exercises during acquisitions and mergers could lead to a better understanding of environmental risks associated to brownfield developments. Mina do Feijão, where the Brumadinho dam was built, was originally part of another company acquired by Vale^5^. A common approach to data description, such as the one published by Rifai and colleagues^4^, could help ensure that data from audits and diligence exercises can be used more effectively and passed more transparently between companies; thereby representing a significant step towards preventing catastrophes from happening.

A number of industries, like aviation, improved safety enormously by establishing rigorous investigation procedures for accidents and sharing outcomes openly. It would, however, be a naïve proposal to ask that accident data be openly shared; as its eventual misuse could have legal and economic implications. On the other hand, open data and metadata initiatives can contribute to a safer and transparent industry, and to the evolution of the fundamental science with positive future impact to sustainable development. Sustainability indexes have provided a good starting point to link relevant practices and some appetite for disclosure to investor’s education and interest^6^. Some level of disclosure of data of key industrial infrastructure could also be captured in sustainability indexes. The use of these data by educators, researchers, and policy makers could contribute to the sustainability of their entire sector.

The resource sector is missing a golden opportunity to learn from their anguish.
